# Ferroptosis and Its Potential Role in Metabolic Diseases: A Curse or Revitalization?

**DOI:** 10.3389/fcell.2021.701788

**Published:** 2021-07-09

**Authors:** Jia-Yue Duan, Xiao Lin, Feng Xu, Su-Kang Shan, Bei Guo, Fu-Xing-Zi Li, Yi Wang, Ming-Hui Zheng, Qiu-Shuang Xu, Li-Min Lei, Wen-Lu Ou-Yang, Yun-Yun Wu, Ke-Xin Tang, Ling-Qing Yuan

**Affiliations:** ^1^National Clinical Research Center for Metabolic Disease, Hunan Provincial Key Laboratory of Metabolic Bone Diseases, Department of Endocrinology and Metabolism, The Second Xiangya Hospital, Central South University, Changsha, China; ^2^Department of Radiology, The Second Xiangya Hospital, Central South University, Changsha, China

**Keywords:** ferroptosis, iron, metabolic diseases, reactive oxygen species, lipid peroxidation

## Abstract

Ferroptosis is classified as an iron-dependent form of regulated cell death (RCD) attributed to the accumulation of lipid hydroperoxides and redox imbalance. In recent years, accumulating researches have suggested that ferroptosis may play a vital role in the development of diverse metabolic diseases, for example, diabetes and its complications (e.g., diabetic nephropathy, diabetic cardiomyopathy, diabetic myocardial ischemia/reperfusion injury and atherosclerosis [AS]), metabolic bone disease and adrenal injury. However, the specific physiopathological mechanism and precise therapeutic effect is still not clear. In this review, we summarized recent advances about the development of ferroptosis, focused on its potential character as the therapeutic target in metabolic diseases, and put forward our insights on this topic, largely to offer some help to forecast further directions.

## Introduction

**FIGURE 1:**
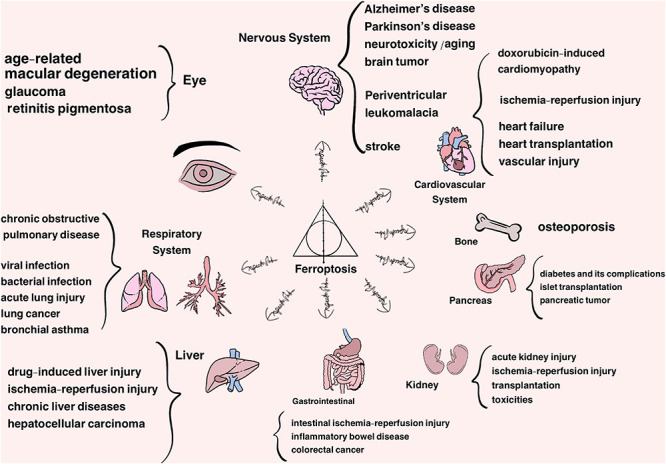
Ferroptosis is approved to participate in the progression of a variety of organs and system diseases, listed above.

Cell death is pivotal for regular growth and development, homeostasis and, to some extent, prevention of diseases (Fuchs and Steller, 2011; Bedoui et al., 2020). Traditionally, cell death is classified into two typical forms, termed as accidental cell death (ACD) (e.g., necrosis) and regulated cell death (RCD) (e.g., apoptosis). RCD is a normal phenomenon that involves exact signaling cascades and mechanisms, taking place in a certain stage of cell life. In comparison, ACD is the process in which cells passively die as a result of infection or injury in most circumstances (Hotchkiss et al., 2009; Fuchs and Steller, 2011; Tang et al., 2019). Along with the thorough study, some other types of RCD have been found gradually over the years, such as necroptosis and autophagy, which are radically different lethal pathways characterized by their distinct features in morphology, biochemistry, molecular mechanisms, among others (Hotchkiss et al., 2009; Denton and Kumar, 2019; Frank and Vince, 2019; Green, 2019; Tang et al., 2020). In-depth research in 2012 defined a new concept known as ‘ferroptosis’ after screening out two compounds, erastin and RSL3, which had a lethal effect on RAS-mutant tumor cells. As a non-apoptotic form of cell death, ferroptosis has its unique trait in morphological, biochemical and genetic aspects, classified as an iron-dependent form of RCD attributed to the accumulation of lipid hydroperoxides and redox imbalance (Dixon et al., 2012; Stockwell et al., 2017). This discovery has become a hot research topic in recent years, and therefore has shed light on the new territory of the progression of diseases, such as neurodegenerative diseases (i.e., Alzheimer’s and Parkinson’s diseases), cardiovascular disease, cancer, ischemia-reperfusion injury, damage of liver and metabolic diseases (Figure 1) and challenges the search for more ways of prevention and treatment (Bruni et al., 2018b; Liang et al., 2019; Weiland et al., 2019; Capelletti et al., 2020; Han et al., 2020; Li J. et al., 2020; Stockwell et al., 2020; Viktorinova and Durfinova, 2021).

Emerging evidence demonstrates that ferroptosis may be part and parcel of the metabolic diseases, or, more specifically, inducing or inhibiting ferroptosis might significant impact these diseases. Thus, in this review, we summarized the recent achievement regarding the pathways controlling ferroptosis and the function of ferroptosis in a series of metabolic diseases.

## Overview of Ferroptosis – Magic of *The Deathly Hallows*

Initially, the compounds erastin and RSL3 were found to induce a diverse phenotype of cell death that can be suppressed by iron-chelating agents (Dolma et al., 2003; Yagoda et al., 2007; Yang and Stockwell, 2008). In 2012, Dixon et al. was the first team ever to denominate the unprecedented topic of ferroptosis, the death type carried out in cells with earmarks of shrinkable mitochondrial volume with lessened mitochondrial crista and denser mitochondrial membrane, but paradoxically with unaltered cell membrane, nucleus and chromatin, on par with other RCD (Yang and Stockwell, 2008; Dixon et al., 2012; Friedmann Angeli et al., 2014). To illustrate, there is an enrichment of reactive oxygen species (ROS) and ferrous ion (Fe2+), accompanied by a biochemical activation of mitogen-activated protein kinases (MAPKs). The bulk of studies to date have clarified that the conventional mechanism of ferroptosis involved the glutathione-glutathione peroxidase 4 (GPX4) axis, namely, restraining the system Xc–, which decreases the ingestion of cysteine, resulting in the deficiency of glutathione (GSH) that leads to the deposition of lipid hydroperoxides, reaching a lethal level (Stockwell et al., 2017, 2020; Chen X. et al., 2020; Tang et al., 2021). Except for the classical channel mentioned above, a significant number of studies mushroomed, revealing that some other pathways also act on the progress of ferroptosis. To date, the requirements for the occurrence of ferroptosis have proven to include iron overload, oxidation of free polyunsaturated fatty acids (PUFAs) and impaired redox pathways, which can be graphically compared to the Elder Wand, the Cloak of Invisibility and the Resurrection Stone, which together make up the Deathly Hallows-Ferroptosis, eventually leading to cell death. Moreover, ferroptosis is regulated by manifold genes, which have already been identified by the shRNA library, but the elaborate procedures still need to be developed (Dixon et al., 2012; Galluzzi et al., 2018; Lei et al., 2019).

### Labile Iron Pool–Powerful Strength of *the Elder Wand*

**FIGURE 2:**
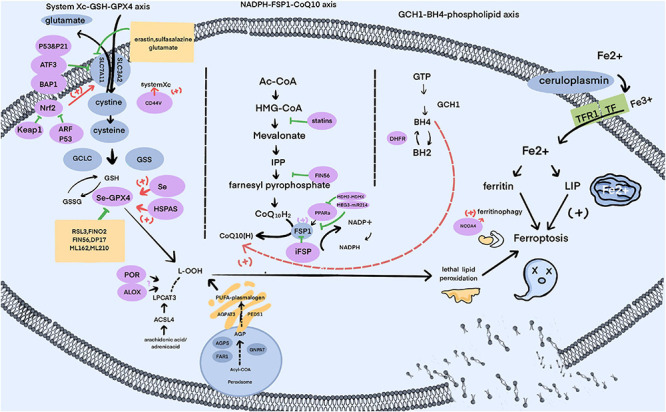
The signal pathways of ferroptosis.

Ferroptosis, as the name suggests, is characterized by the need for iron. In the body, Fe2+ can be transformed to Fe3+ by ceruloplasmin, resulting in the combination of Fe3+ with transferrin (TF) as a complex endocytosed through membrane protein TF receptor 1 (TFR1) (Li J. et al., 2020; Zheng and Conrad, 2020). Intracellular Fe3+ is reduced to Fe2+, either used to compose iron-dependent enzymes (Anderson and Vulpe, 2009) or stored in the labile iron pool (LIP) and ferritin (Bogdan et al., 2016), and the redundancy exported by ferroportin 1 (FPN1), multi-copper ferroxidase (e.g., ceruloplasmin) and ion transporter, lipocalin 2 (LCN2) (Bogdan et al., 2016; Anderson and Frazer, 2017; Li J. et al., 2020; Liu J. et al., 2021). Ultimately, the increased iron released to the LIP by ferritin-targeted autophagy, depletion of ferritin or some other circumstances is the essential condition for ferroptosis, just like a wizard’s wand, waiting for a killing curse to taste death (Figure 2).

Ferroptosis can be obstructed by iron chelators (Friedmann Angeli et al., 2014), knockdown of TFR1 (Gao et al., 2015; Torii et al., 2016) (identified as a ferroptosis marker) (Feng et al., 2020) and eliminating autophagy-related genes or the selective cargo receptor nuclear receptor coactivator 4 (NCOA4) for blocking ferritinophagy (Gao et al., 2016; Zheng and Conrad, 2020). The stress response gene, transcriptional regulator (NUPR1), targeted LCN2 as an effector gene to inhibit iron-dependent ferroptosis and LCN2 can retard acute pancreatitis by lowering the iron level in the cytoplasm (Liu J. et al., 2021), which help us draw inspiration from this pathway to explore the relationship between iron metabolism and ferroptosis on the gene level. Lately, an unanticipated axis of ATM (Ataxia-Telangiectasia)-MTF1 (metal-regulatory transcription factor 1)-Ferritin/FPN1 contributed to lessen labile iron via inhibiting ATM, which was detected by P. H. Chen et al., discovering a new dimension in ferroptosis through kinome screen (Chen P. H. et al., 2020). In contrast, decreasing the expression of ferritin (Chen P. H. et al., 2020), FPN1 (Bao et al., 2020; Chen P. H. et al., 2020) and ceruloplasmin (Shang et al., 2020) have testified to be sensitized to ferroptosis. Other iron-related proteins, such as poly-(rC)-binding protein 1 (PCBP1) (Protchenko et al., 2020; Zheng and Conrad, 2020) and heme oxygenase-1 (HO-1) (Adedoyin et al., 2018; Fang et al., 2019), have been found to be involved in ferroptosis as well. A recent study supported that ferritin-containing multivesicular bodies and exosomes are qualified to discharge iron driven by prominin2, illuminating a new mode to prevent ferroptosis (Brown et al., 2019). What merits attention is that the endothelial cell-secreted exosome has proven to throw a wrench in the glucocorticoid-induced osteoporosis process by abating ferritinophagy (Yang et al., 2021). Dysregulation of iron homeostasis and metabolism is substantiated to have a close connection to diverse metabolic diseases, such as diabetes (Dubey et al., 2020), obesity, metabolic syndrome (Gonzalez-Dominguez et al., 2020), osteoporosis (Che et al., 2020; Sato et al., 2020) and AS (Wunderer et al., 2020). Thereby, we forecast that the link between exosome and iron metastasis in ferroptosis may be a heated discussion in the near future. Likewise, exosomes as a therapy method against ferroptosis in varied metabolic diseases may also soon invigorate a conversation.

### Oxidation of PUFAs – Death Clothed in *the Cloak of Invisibility*

According to a series of studies, free PUFAs are known to be the imperative substrates of lipid oxidation, which are esterified into membrane phospholipids and result in an oxidation that triggers ferroptosis (Yang and Stockwell, 2016; Doll et al., 2019; Zheng and Conrad, 2020), likened to the attire in the Cloak of Invisibility toward death. Phosphatidylethanolamine (PE) containing arachidonic acid (AA) and adrenaline is certified to initially be this kind of phospholipid (Kagan et al., 2017). The substrates mentioned above are catalyzed by Acyl-CoA synthetase long-chain family member 4 (ACSL4) (Kuch et al., 2014) and lysophosphatidylcholine acyltransferase 3 (LPCAT3) (Kagan et al., 2017; Zheng and Conrad, 2020) into their acyl-CoA esters and lysophospholipids. The next steps in the pathway of lipid oxidation-induced ferroptosis are still not yet interpreted perspicuously, in that lipoxygenase (LOX), especially ALOX15, has been widely reported to actuate lipid oxidation (Yang et al., 2016; Kagan et al., 2017; Wenzel et al., 2017; Zheng and Conrad, 2020). On the other hand, several researches have developed, explaining that the knockdown Alox15 gene cannot succeed to reverse ferroptosis induced by GPX4 deficiency in multiple types of cells (Brutsch et al., 2015; Matsushita et al., 2015) and the expression of ALOX (mRNA level) remains at a low content in many cancer cells (Zou et al., 2020b). Clearly speaking, lipid autoxidation may be the primary cause of ferroptosis (Shah et al., 2018). Nonetheless, it is worth noting that cytochrome P450 oxidoreductase (POR), located in the endoplasmic reticulum, conduces to phospholipid peroxidation (Zou et al., 2020b) and leads to membrane damage, with the participation of cytochrome b5 reductase (CYB5R1) (Yan et al., 2021). This evidence may highlight the importance of enzymatic reactions in ferroptosis, given that with the effect of enzymes, the phospholipid hydroperoxide (PLOOH) threshold may be reached physiologically, in spite of the antioxidant systems (Shah et al., 2018).

Apart from the process discussed above, another pathway of lipid metabolism in ferroptosis has emerged, which is an ACSL4/LPCAT3-independent way that relies on peroxisomes, whose function is to synthesize plasmalogens, an alternative substrate of lipid oxidation classified as a subclass of ether phospholipids (Zou et al., 2020a). This pathway is indebted to the peroxisomal enzymes involved: alkylglycerone phosphate synthase (AGPS), fatty acyl-CoA reductase 1 (FAR1), and glyceronephosphate O-acyltransferase (GNPAT), under which the precursor 1-O-alkyl-glycerol-3-phosphate (AGP) is synthesized and subsequently transported to the endoplasmic reticulum to form PUFA-plasmalogen by 1-acylglycerol-3-phosphate O-acyltransferase 3 (AGPAT3) and endoplasmic reticulum(ER)-resident enzyme plasmanylethanolamine desaturase 1 (PEDS1) (Tang and Kroemer, 2020; Zou et al., 2020a). Therefore, insights into the peroxisomes may provide a newly built framework of the development of ferroptosis, which may guide a direction to treatment; meanwhile, complete access to this pathway and the other roles the peroxisomes play in ferroptosis remain to be tested.

### Three Pathways – The Repair Network of *the Resurrection Stone*

There are three predominant pathways, similar to the resurrection stone in Harry Potter, which manage to repair the peroxide state, contributing to prevent ferroptosis and reviving cells in the body. By blocking these procedures, cells may be exposed to the sensitivity of ferroptosis.

#### System Xc-GSH-GPX4 Axis

The amino acid system Xc-GSH-GPX4 axis is an indispensable part of lipid peroxidation elimination. Comprised of SLC3A2 and SLC7A11, system Xc– imports cystine into cells by reversing glutamate transport (Wang et al., 2020b; Li N. et al., 2021). Moreover, cystine transforms into cysteine, performing as raw material in the synthesis of GSH. As the glutathione peroxidase, the selenoprotein GPX4 catalyzes the combination of lipid hydroperoxides (L-OOH) with the sulfhydryl group of reduced glutathione, converting the harmful substances into non-toxic lipid alcohols (L-OH) and hence blocking the ROS chain reaction, avoiding ferroptosis (Yang et al., 2014; Ingold et al., 2018; Seibt et al., 2019). The aforementioned contents are the main framework of the system Xc-GSH-GPX4 axis, and plenty of molecules are certified to be mediators of the above substances, which primely enrich the pathway described below.

To regulate system Xc–. (Erythoid-derived)-like 2 (Nrf2) belongs to the cap-n-collar subfamily of transcription factors, proven to be conjugated with Kelch-like ECH-associated protein 1 (Keap1) in non-oxidizing states and while the oxidative stress was aggrandized, it may separate from Keap1, acting as a key member in oxidation-reduction reactions. According to manifold studies, the Nrf2-Keap1 pathway is able to give an impulse to system Xc–, enhancing the resistibility of ferroptosis via Nrf2 overexpression or Keap1 drawdown, which was further confirmed to occur in the development of retinopathy in Type 1 diabetes (T1DM) (Fan et al., 2017; Carpi-Santos and Calaza, 2018). Notably, active Nrf2 is in a position to ease delayed gastric emptying in obesity-induced diabetic (Type 2 diabetes, T2DM) mice, implying a potent role of this pathway in diabetes (Sampath et al., 2019). In addition, an alternative reading frame product of the CDKN2A locus (ARF) can weaken the course of Nrf2-controlled sensitization of SLC7A11, taking a role in facilitating ferroptosis by relying or not relying on tumor suppressor gene p53 in cancer cells (Chen et al., 2017). Based on previous data, p53 is crucial for ferroptosis by performing double- action work: boost ferroptosis on SLC7A11 expression (L. Jiang et al., 2015; Liu J. et al., 2020) and suppress ferroptosis by declining susceptibility, which may need a share with its target gene, P21 (Liu J. et al., 2020; Venkatesh et al., 2020b). Furthermore, activating transcription factor 3 (ATF3) is also proven to couple with a SLC7A11 promoter to advance ferroptosis in a p53-independent way, as with the tumor suppressor BRCA1-associated protein 1 (BAP1) (Zhang Y. et al., 2018; Zhang et al., 2019a; Wang et al., 2020b). Several factors, such as glutamate, sorafenib, sulfasalazine, imidazole ketone erastin, diaryl-isoxazoles, and INF-γ, are also approved to be inhibitors of system Xc–, competitively or not (Dixon et al., 2012, 2014; Newell et al., 2014; Leu et al., 2019; Zhang X. et al., 2019; Zitvogel and Kroemer, 2019).

In metabolic and endocrine diseases, eliminating SLC7A11 is related to inhibiting the growth of pancreatic ductal adenocarcinoma (Badgley et al., 2020). In addition, researchers have identified a gene, pyruvate dehydrogenase kinase 4 (PDK4), that is obligated to alter ferroptosis sensitivity in human pancreatic ductal carcinoma cells through the system Xc–, influenced by glucose metabolism, implicating a new view of cancer therapy (Song et al., 2021). Of note, lowering the level of CD44 variant (CD44v), which can steady the system Xc–, increases insulin secretion and contributes to the amino acid transport regulated by L-type amino acid transporter LAT1 in pancreatic β cells; thus guiding a new therapeutic target in diabetes (Kobayashi et al., 2018).

To regulate GPX4. GPX4 is designated as a determinant upstream mediator of ferroptosis, extensively existing in cytoplasm, the cell nucleus, mitochondria and other cellular organs (Arai et al., 1996; Friedmann Angeli et al., 2014; Seibt et al., 2019; Li N. et al., 2021). RSL3 was discovered to be an inhibitor of GPX4 to induce ferroptosis sufficiently and early (Yang et al., 2014). Subsequently, compounds FINO2, FIN56, ML162, ML210, DPI7, DPI10 and buthionine sulfoximine, which have the same effect as RSL3, have been discovered as well (Gaschler et al., 2018; Seibt et al., 2019; Li N. et al., 2021). The latest research depicted that folate-vectorized exosomes loaded with erastin were capable of inhibiting GPX4 in triple-negative breast cancer cells (Yu et al., 2019), also emphasizing the crosstalk between exosome and ferroptosis. Currently, the molecular mechanism that selenium takes a part of in the synthesis of GPX4 by shaping into the 21st amino acid, selenocysteine (Sec), has been enucleated. This gives insight into the point that using selenium is necessary to avoid ferroptosis due to its ability to activate the transcription factors TFAP2c and Sp1, causing the reinforcement of GPX4, and resulting in the safeguarding of neurons (Ingold et al., 2018; Alim et al., 2019). Analogously, the mevalonate (MVA) pathway subserves the selenocysteine tRNA in order to participate in the synthesis of GPX4, and isopentenyl pyrophosphate (IPP) and COQ10 are the main products of this process (Warner et al., 2000). Notably, the heat shock protein family also affects GPX4; specifically, HSPA5 attenuates erastin-induced GPX4 evanishment while the chaperone-mediated autophagy on the strength of HSP90 antagonizes that matter, possibly hastened by legumain (Zhu et al., 2017; Wu Z. et al., 2019; Chen et al., 2021).

Intriguingly, as the micronutrient, selenium (Se) and selenoprotein are integral in several metabolic and endocrine diseases, such as thyroid disease and diabetes, and raising their level has been relevant to insulin-induced, hydrogen peroxide (H2O2)-dependent signaling impairment, resulting in insulin resistance and hyperglycemia (Wang et al., 2016; Kim et al., 2019; Schomburg, 2020). Meanwhile, in the Se-deficient population, serum Se positively correlates with glucose, indicating that Se supply is basilical for glucose homeostasis (Wang et al., 2020g). It is conceivable, but not yet demonstrated penetratingly, that ferroptosis could be significantly diminished by mediating GPX4 with a supplement of Se, playing a role in insulin regulation and diabetes.

#### NADPH-FSP1-CoQ10 Axis

Ferroptosis suppressor protein 1 (FSP1) (previously referred to as apoptosis-inducing factor mitochondrial 2, AIFM2) was one of anti-ferroptotic genes identified relatively recently (Stockwell et al., 2020). By means of its catalytic action with nicotinamide adenine dinucleotide phosphate (NADPH), CoQ10 (also referred to as ubiquinone) regenerates so as to exhibit oxidation resistance, capturing lipid peroxyl radicals to suppress ferroptosis. Correlation studies examined this pathway, giving rise to an unaided parallel route of ferroptosis distinct from the foregone system Xc-GSH-GPX4 axis. FSP1-induced ferroptosis resistance is corroborated to take place in a number of cancer cell lines, elucidating a new anticancer target (Elguindy and Nakamaru-Ogiso, 2015; Bersuker et al., 2019; Doll et al., 2019). However, whether upregulating FSP1 can really enshield cells not to suffer from ferroptosis remains to be clarified in the near future (Santoro, 2020). It is worth noting that a study recently revealed that FSP1 could solely depress erastin-, RSL3-, and sorafenib-induced ferroptosis without CoQ10, dealing with the endosomal sorting complexes required for transport (ESCRT)-III–dependent membrane repair (Dai et al., 2020). Nonetheless, this research did not discover the downstream target of FSP1, and this still awaits us and requires a deeper investigation into the mechanism.

To regulate this axis. Murine double minute 2 (MDM2) family (MDM2 and MDMX) are regarded as negative adjusters of p53. Inhibiting the MDM2-MDMX complex was clarified to heighten the levels of FSP1 protein by remodeling peroxisome proliferator-activated receptor α (PPARα) activity (Venkatesh et al., 2020a). It is noteworthy that FIN56 initiates ferroptosis by way of depleting GPX4 and the mevalonate-derived CoQ10 (Shimada et al., 2016; Stockwell et al., 2020). By the same token, statins could bridle CoQ10 for being a hindrance of the enzyme HMG CoA reductase (Shimada et al., 2016). Strikingly, the long non-coding RNA maternally-expressed gene 3 (MEG3)-microRNA-214 (miR-214) axis may negatively regulate FSP1, which was known to direct against activating transcription factor 4 (ATF4, a SLC7A11 promoter) (Bai et al., 2020b). cAMP-response-element-binding protein (Nguyen et al., 2020) and P53 (Bersuker et al., 2019; Doll et al., 2019) also have a share in FSP modulation.

In cellular metabolism, FSP1 was estimated to accelerate glycolysis via oxidizing NADH in the outer side of the mitochondrial inner membrane in brown adipose tissue (BAT), resulting in retarding diet-induced obesity and insulin resistance (Nguyen et al., 2020). Replenishing mitochondrial CoQ has been showed to ameliorate insulin resistance in adipocytes, principally by decreasing superoxide/H2O2 production via complex II (Fazakerley et al., 2018). An interesting research reported there might be a marginal coenzyme Q10 deficiency in athletes, which was associated with blood glucose and antioxidant capacity (Ho et al., 2020) and then through recharging ubiquinol, resulting in exercise performance upgrades with a raised level of free fatty acids (FFA) (Chen et al., 2019). Besides, CoQ10 united with pioglitazone modified the mRNA expression of adipocytokines and oxidative stress parameters in diabetic rats (Maheshwari et al., 2020); meanwhile, CoQ10 with alpha lipoic acid (ALA) could decrease degeneration and apoptosis of dorsal root ganglion (DRG) neurons of diabetic neuropathy (DN), seemingly by mediating the expression of caspase 3 and uncouplingprotein 2 (UCP2) proteins (Sadeghiyan Galeshkalami et al., 2019). In summary, abundant investigations have documented that supplementation of CoQ may have a beneficial effect in metabolic syndrome (Raygan et al., 2016; Mazza et al., 2018), diabetes (Zhang S. Y. et al., 2018) and its complications (Mantle, 2017), such as diabetic retinopathy (Zhang X. et al., 2017; Derosa et al., 2019), macroangiopathy (disorder of lipid metabolism and AS) (Montano et al., 2015; Zhang P. et al., 2018; Suarez-Rivero et al., 2019), and diabetic kidney diseases (Zhang X. et al., 2019). Yet, there is a requirement for more participant-involved studies and clinical trials to ravel the molecular mechanism and ensure the therapeutic effect is meeting the clinical needs. We speculate that ferroptosis could be attached to these morbid processes, and its inducers and inhibitors targeted to the NADPH–FSP1-CoQ10 axis may be useful for treatment, although further study is required to test this possibility.

#### GCH1-BH4-Phospholipid Axis

Recently, with a method of genome-wide activation screen, Kraft et al. (2020) documented that GTP cyclohydrolase-1 (GCH1) and its metabolic derivatives tetrahydrobiopterin/dihydrobiopterin (BH4/BH2) partake in developing ferroptosis resistance. Mechanistically, BH4 is capable of selectively deterring autoxidation of phospholipids with two polyunsaturated fatty acyl tails and expediting the generation of CoQ10 (Kraft et al., 2020), in order to endowBH4 with emerging lipophilic radical-trapping antioxidants (RTAs) requiring dihydrofolate reductase (DHFR) for rebirth (Soula et al., 2020). Until recently, the discussions about this axis were incomplete; therefore, more in-depth exploration of its pathophysiological role in ferroptosis needed to be conducted.

Accumulating evidence illustrate that the GCH1-BH4-phospholipid axis plays a part in energy metabolism and metabolic diseases (Kim and Han, 2020). Concretely, BH4 is signalized for being a cofactor involving in the enzymatic conversion of amino acids; for instance, tyrosine and phenylalanine to precursors of dopamine and serotonin, as well as the formation of nitric oxide (NO) demand for BH4 combining with nitric oxide synthase (NOS) (Mayer and Werner, 1995; Kim and Han, 2020). All of the biological functions are the cornerstone for BH4’s role in glycolipid metabolism disorder, endothelial injury and inflammation. BH4 decreased due to oxidative stress, opening the door for BAT dysfunction, mainly through NO and noradrenaline signaling, contributing to higher obesity, insulin resistance, and glucose intolerance (Oguri et al., 2017). In addition, in hypercholesterolemia, oxidized low-density lipoproteins (ox-LDLs) reduce mRNA expression of GCH1 and BH4, leading to a decline of NO damaging the endothelium to induce vascular injury (i.e., AS) (Douglas et al., 2018; Kim and Han, 2020), which may be accelerated by nicotine (Li et al., 2018). Interestingly, more studies were performed to show that GCH1/BH4 may act as a therapeutic target for diabetic cardiomyopathy (Wu et al., 2016; Kim et al., 2020; Carnicer et al., 2021). Also, CTRP13 (Wang et al., 2020f), zinc (Liu P. et al., 2020), and curcumin nanoparticles (Abu-Taweel et al., 2020) have been utilized to exalt the level of GCH1 (Alp et al., 2004) or BH4 (Shah et al., 2017), resulting in observation of a relief of endothelial dysfunction. These results furnished a clue that the ferroptosis appears to assist the metabolic and vascular diseases mentioned before dependency on GCH1/BH4, and repressed ferroptosis may protect cells from the harm of those pathological processes, which may be a hotspot for the further investigation. Strikingly, a new study unveiled that glucose starvation-induced energy stress significantly attenuated erastin- and cysteine/GPX4 depletion-induced ferroptosis due to AMP-activated protein kinase (AMPK) in immortalized mouse embryonic fibroblasts (Lee et al., 2020); whereas, activation of AMPK is verified to recede T2DM-induced BH4 reduction by preventing GCH1 degradation, further intimating about the potential role of ferroptosis in metabolic diseases (Day et al., 2017).

## Ferroptosis Cast Spells to Metabolic and Endocrine Diseases

### Ferroptosis and Diabetes and Its Complications

According to the investigation of the International Diabetes Federation, under half a billion people are suffering from diabetes worldwide and this number is increasing rapidly (Saeedi et al., 2019). Diabetes is classified into two types: T1DM, caused by damnification of pancreatic β cells, along with insufficient insulin secretion, and T2DM, which is mainly induced by insulin resistance (Saberzadeh-Ardestani et al., 2018; Galicia-Garcia et al., 2020). Both types contribute to the building of a high glucose state, which may inflict SLC7A11 and SLC3A2L impairment, leading to system Xc– dysfunction (Koppula et al., 2017). It has been affirmed that patients with T2DM are devoid of GSH, especially if microvascular complications are present (Lutchmansingh et al., 2018). Substantial researches have called attention to the close relationship between iron and glucose metabolism; that is, iron deficiency and excess may affect glucose regulation (Fernandez-Real et al., 2015), and high glucose may give rise to iron overload (Shu et al., 2019), which is known to trigger ferroptosis. Meanwhile, under iron overload condition, insulin resistance might be brought on by oxidative stress (Sung et al., 2019) and the pancreatic fat fraction has proven to be associated with glucose dysregulation (Shur et al., 2020). Evidence has shown that ferroptosis arises in patients with diabetes and its complications and inhibiting it may become a welcome sign in treatment.

#### Diabetes and Pancreatic Dysfunction

The damage of β cells in T1DM mainly occurs due to the negative effect of proinflammatory cytokines and its products: reactive oxygen and nitrogen species (RNS) (Lenzen, 2017). A study conducted in 2018 has indicated that ferroptosis-inducing agents (FIA), such as erastin or RSL3, have the ability to impact islet function *in vitro* (Bruni et al., 2018b) and ferroptosis may be involved in islet isolation and transplantation (Bruni et al., 2018a). β cells often have decreased levels of H2O2-detoxifying enzymes, i.e., catalase, glutathione peroxidase 1 (Lenzen, 2008), GPX7 and GPX8 (Mehmeti et al., 2017), as well as the generation of ROS and iron accumulation (Krummel et al., 2021), owing to proinflammatory cytokines (Lortz et al., 2014). Krummel et al. (2021) determined that β cells may have a higher sensitivity to ferroptosis and confirmed that GPX4 distributes throughout the β cells to a large extent, eliminating that GPX4 induces ferroptosis. Interestingly, proinflammatory cytokine-induced death is independent of ferroptosis, probably because the offspring of toxic cytokines, nitric oxide, can wipe out generated lipid radicals in the membrane.

Although the phenotype and mechanism of ferroptosis developed in β cells have been pressed for in-depth exploration, some substances directed against ferroptosis have been found to ameliorate impairment of transplanted islet cells and treat diabetes. Bilirubin may protect the islet by raising GPX4, upregulating Nrf2/HO-1 and chelating iron to interdict ferroptosis (Yao et al., 2020). Cryptochlorogenic acid (CCA), the active constituent of the mulberry leaf, was reported to target some major regulators (system Xc-GPX4, Nrf2 and NCOA4) to inhibit ferroptosis in a concentration-dependent manner in a diabetic model (Zhou, 2020). Similarly, in T2DM, a natural iron chelator, quercetin, may also exert a positive effect to reverse pancreatic iron deposition as an inhibitor of ferroptosis (Li D. et al., 2020). Additionally, as a high-risk factor for pancreatic dysfunction and T2DM (Grau-Perez et al., 2017), chronic arsenic exposure may induce islet autophagy (Wu et al., 2018) and impact insulin secretion in pancreatic β cells for its destructive effect in mitochondrial metabolism (Dover et al., 2018; Carmean et al., 2019; Zhang Q. et al., 2019). One research study demonstrated that ferroptosis can be triggered by arsenic, which causes mitochondrial ROS-dependent autophagy via regulating iron level in a MIN6 cell model (Wei et al., 2020). All results clearly showed that inhibiting ferroptosis may expect to improve islet viability, and propose new therapeutic targets. However, many questions remained unanswered: can blocking ferroptosis truly increase the lifespan of a transplanted islet?; which factors or pathways may trigger ferroptosis under physiopathologic conditions?; and what is the integrated molecular mechanism? The answers to these questions still need to be worked out, but will not obstruct expectations of the underlying role of ferroptosis in diabetes.

#### Diabetic Nephropathy (DN)

Until fairly recently, researchers demonstrated that kidney tubular cell death in DN is related to ferroptosis based on the observation of declining expression of system Xc– and GPX4 mRNA and the enhancive ROS and lipid oxidation *in vivo* and *in vitro* respectively, which can be ameliorated by ferrostatin-1 (Fer-1) (Wang et al., 2020e; Kim et al., 2021; Li S. et al., 2021), implicating the positive curative effect to hinder ferroptosis. A recent study revealed the mechanism, which may have evinced HO-1 regulated by hypoxia-inducible factor (HIF), administering to iron accumulation by decomposing heme, resulting in the induction of ferroptosis to harm renal tubular in db/db mice (Feng et al., 2021), but detailing the pathophysiological process merits further investigation. High-mobility group box-1 (HMGB1) is a transcription factor enriched in the cell nucleus, involved in DNA repair and synthesis of inflammatory factors by activating the NF-κB signaling pathway (Xu T. et al., 2019). It exerted an effective effect to impede the development of DN via suppressing HMGB1 (Chen et al., 2018), by which it may inhibit high glucose-induced activation of TLR4/NF-κB and ferroptosis in mesangial cells through the Nrf2 pathway (Wu et al., 2021). Controversially, Nrf2 has a dual effect in diabetes and its complications, such as DN (Xu J. et al., 2012; Uruno et al., 2013), and upregulation of Nrf2 appears to block ferroptosis to delay the progression of DN (Li S. et al., 2021) while it may also attenuate DN by augmenting the expression of intrarenal angiotensin-converting enzyme-2 and angiotensin 1–7 receptor (Zhao et al., 2018). These present researches indicate the significance of Nrf2 preliminarily; therefore, the ferroptosis-specific role of Nrf2 in DN should be further elucidated.

#### Diabetic Myocardial Dysfunction

Myocardial oxidative stress and fibrosis produced by high glucose arethe major causes of diabetic cardiomyopathy (DCM) (Zang et al., 2020). Emerging evidence has manifested that suppressing ferroptosis is ofbenefit in delaying the progression of DCM. Firstly, GPX4 has beenobserved to ameliorate streptozotocin-associated cardiac injury(Baseler et al., 2013) and its shortage, related to mitochondrial lipidperoxidation and resulting in cardiac hypertrophy in mice under a diet high in sugar and fat (Behring et al., 2014). In addition, ferroptosis inhibitors, vitamin E (Shirpoor et al., 2009) and CoQ10 (Huynh et al., 2012), have been reported to improve cardiac diastolic dysfunction in diabetic models successively. Heat shock factor 1 (HSF1) has recently been shown to be able to mitigate palmitic acid-induced lipid peroxidation in obesity and T2DM-involved cardiomyopathy and further mediate the transcription of iron metabolism-related genes (e.g., Tfrc, Fth1, and Slc40a1) to maintain iron homeostasis in H9c2 cardiomyoblasts (Wang et al., 2021). We therefore believe that links between ferroptosis and diabetic cardiomyopathy should exist. Remarkably, and similar to the previous section on DN, Nrf2 also became a central issue by academicians for its protective role to cardiac cells in both T1DM and T2DM, principally via the Nrf2-Keap1 pathway (Ge et al., 2019) and its detrimental role noticed in fibroblast growth factor 21-knockout mice (Yan et al., 2015). A novel experiment uncovered that in diabetic setting, cardiac autophagy is disserved, causing cell death and myocardial damage, mainly due to ferroptosis triggered by the activation of Nrf2 (Zang et al., 2020). Thus far, there have been many drugs found to target the key regulators of ferroptosis to resist ROS generation and lipid peroxidation in DCM. Wang et al. (2020f) noted that exogenous spermine may upregulate calcium-sensitive receptor (CaSR) expression by blocking the Nrf2-ROS-p53-muscle-specific ring finger protein 1 (MuRF1) in T1DM rats, ultimately recuperating calcium homeostasis and decreasing oxidative stress. Sulforaphane (SFN) has been found to be capable of activating Nrf2 through AMPK/AKT/GSK-3β signaling pathways in order to upregulate its downstream metallothionein (MT), which is a set of low molecular weight proteins enriched with cysteine, resulting in reversing oxidative damage and fibrosis (Gu et al., 2017). Confirmed recently, this entire protective process depends on the AMPK partaking (Sun et al., 2020). Other compounds that play similar roles are listed in Table 1. Despite this, it remains to be clarified whether these compounds exert a protective effect by interrupting ferroptosis in diabetic cardiac cells.

**TABLE 1:** Emerging compounds targeted key regulators of ferroptosis to attenuate diabetic myocardial dysfunction.

**Type of disease**	**Compounds**	**Effects**	**Mode of action**	**Possible role in ferroptosis**	**Animal models**	**References**
Diabetic cardiomyopathy (DCM)	Exogenous spermine	Attenuate DCM Reduce fibrosis Relieve oxidative stress Upregulate myocardial membrane CaSR	Block Nrf2-ROS-p53-MuRF1 axis	Inhibitor	T1DM rats	Wang et al., 2020f
	Sulforaphane	Attenuate DCM Reduce fibrosis Relieve oxidative stress Prevent hypertrophy	Facilitate Nrf2-metallothionein pathway through AMPK/AKT/GSK-3β signaling; Mitigate the diabetes-induced inhibition of LKB1/AMPK/sirtuin 1/PGC-1α signaling	Inhibitor	T2DM mice	Gu et al., 2017; Sun et al., 2020; Zhang et al., 2014
	Empagliflozin	Attenuate DCM Reduce fibrosis Relieve oxidative stress Meliorate myocardial structure and function	Facilitate Nrf2/ARE signaling Suppress TGF-β/Smad pathway	Inhibitor	T2DM mice	Li L. et al., 2019
	Fibroblast growth factor-21	Prevent DCM Relieve oxidative stress Weaken lipotoxicity	Facilitate AMPK-AKT2-NRF2-mediated antioxidative pathway and AMPK-ACC-CPT-1-mediated lipid-lowering pathway	Inhibitor	T2DM mice	Yang et al., 2018
	Allopurinol	Attenuate DCM Relieve oxidative stress Ameliorate autophagy over-activation	Facilitate Nrf2/p62 signaling	Inhibitor	T1DM rats	Luo et al., 2020
	Luteolin	Attenuate DCM Relieve oxidative stress Ease inflammatory responses	Facilitate NF-κB pathway and improve Nrf2 expression	Inhibitor	T1DM rats	Li L. et al., 2019
	Sirt6&Sirt3 (nuclear and mitochondrial sirtuins)	Prevent DCM Relieve oxidative stress Avert insulin resistance of cardiomyocytes	Sirt3 relieves oxidative stress to maintain Sirt6 levels Sirt6 stimulates Nrf2-dependent Sirt3 gene transcription	Inhibitor	db/db mice	Kanwal et al., 2019
	Piceatannol; dimethyl fumarate	Attenuate DCM Relieve oxidative stress Ease inflammatory responses	Facilitate Nrf2/HO-1 pathway	Inhibitor	T1DM rats	Hu et al., 2018; Li H. et al., 2019
	Broccoli sprout extract; Zinc; Saxagliptin; aspalathin	Attenuate DCM Relieve oxidative stress Ease inflammatory responses	Facilitate Nrf2/HO-1 pathway	Inhibitor	db/db mice	Dludla et al., 2017; Wang et al., 2017; Xu et al., 2016; Zhang L. et al., 2020
	Bailcalin; pterostilbene	Prevent DCM Relieve oxidative stress Weaken lipotoxicity Reduce fibrosis and hypertrophy	Facilitate AMPK/Nrf2/HO-1 axis for improving nuclei translocation of Nrf2	Inhibitor	T2DM mice	Kosuru et al., 2018; Li R. et al., 2019
	Resveratrol	Attenuate DCM Relieve oxidative stress Improve mitochondrial function Reduce ventricular hypertrophy and myocardial fibrosis	Activate SIRT1 to facilitate PGC-1α deacetylate, increasing expression of NRF-2	Inhibitor	T1DM mice and SIRT1KO mice	Ma et al., 2017
	Klotho	Attenuate DCM Relieve oxidative stress Repress cardiac inflammatory cytokines Prevent cardiac cell remodeling	Inhibit NF-κB activation and Improve Nrf2 expression	Inhibitor	T1DM rats	Guo et al., 2018
	Bakuchiol	Attenuate DCM Relieve oxidative stress Reduce fibrosis and hypertrophy	Facilitate SIRT1-Nrf2 signaling pathway to block TGF-β1/Smad3 signaling activation	Inhibitor	STZ-treated mice	Ma et al., 2020
	Isoliquiritigenin	Attenuate DCM Relieve oxidative stress Reduce cardiac apoptosis, fibrosis, and hypertrophy	Suppress MAPKs expression and facilitate Nrf2 signaling pathway	Inhibitor	T1DM mice	Gu et al., 2020
	Obeticholic acid	Attenuate DCM Relieve oxidative stress Repress cardiac inflammatory cytokines Reduce fibrosis	Regulate FXR/Nrf2 signaling	Inhibitor	db/db mice	Wu et al., 2019
	Phloretin	Attenuate DCM Relieve oxidative stress Reduce fibrosis	Facilitate the dissociation of Keap1/Nrf2 Complex to promote Nrf2 expression	Inhibitor	STZ-treated mice	Ying et al., 2018
	Cyclovirobuxine D	Attenuate DCM Relieve oxidative stress Resume mitochondrial membrane potential	Upregulate Nrf2-NQO-1/Prdx1(both are downstream defense genes of Nrf2) signaling pathway	Inhibitor	T2DM mice	Jiang et al., 2020
	Thymoquinone	Attenuate DCM Relieve oxidative stress Repress inflammatory reaction	Increase Nrf2 expression decrease iNOS and improve EPO and VEGF	inhibitor	STZ-treated mice	Atta et al., 2018
	Andrographolide	Attenuate DCM Relieve oxidative stress Repress inflammatory reaction Prevent cardiac cell remodeling promote the parameters of cardiac function	Repress NOX and enhance Nrf2 expression Ameliorate NF-κB-mediated inflammatory reaction	Inhibitor	STZ-treated mice	Liang et al., 2018
	Syringaresinol	Attenuate DCM Relieve oxidative stress Repress inflammatory reaction Reduce fibrosis	Downregulate Keap1 expression to promote Nrf2-NQO-1/HO-1 pathway	Inhibitor	T1DM rats	Li G. et al., 2020
	Myricitrin	Attenuate DCM Relieve AGEs-induced oxidative stress and inhibit mitochondrial injury and apoptosis Repress cardiac inflammatory cytokines	Facilitate the phosphorylation of AKT and GSK-3β, activate Nrf2-NQO-1/HO-1 signaling; block IκBα/NF-κB pathway	Inhibitor	STZ-treated mice	Liao et al., 2017; Zhang et al., 2017
	LAZ3	Attenuate DCM Relieve oxidative stress Prevent cardiac cell remodeling	Downregulate miR-21 expression, promoting PPARa/NRF2 signaling	Inhibitor	STZ-treated mice	Gao et al., 2018
	PPARa	Aggravate oxidative stress and cardiac injury of DCM	Silencing PPARa may suppress HMGCS2 expression, resulting in facilitating NRF2/ARE signaling pathway	Inducer	STZ-treated mice	Wang et al., 2020c
	Notoginsenoside R1	Attenuate DCM Relieve oxidative stress Reduce apoptosis, serum lipid levels, and insulin resistance Promote left ventricular function	Improve ERα level, resulting in facilitating AKT-Nrf2 signaling	Inhibitor	db/db mice	Zhang et al., 2018b
	Adiponectin	Attenuate DCM Relieve oxidative stress Reduce apoptosis Promote cardiac function	Facilitate Nrf2 and Brg1 level jointly to improve HO-1 expression	Inhibitor	STZ-treated mice	Li et al., 2015
Myocardial ischemia/reperfusion injury (MIRI)	Honokiol	Ameliorate MIRI Relieve oxidative stress Reduce apoptosis Lessen the infarct size	Facilitate SIRT1-Nrf2 signaling pathway	Inhibitor	T1DM rats	Zhang et al., 2018a
	Resveratrol	Ameliorate MIRI Relieve oxidative stress	Upregulate SIRT1 and downregulate GSK3β to promote Nrf2 expression	Inhibitor	T2DM mice	Xu G. et al., 2019
	Geniposide	Ameliorate MIRI Relieve oxidative stress Reduce apoptosis	Facilitate Nrf2/HO-1 pathway	Inhibitor	T2DM mice	Wang et al., 2019
	Luteolin	Ameliorate MIRI Relieve oxidative stress Increase cardiac tissue viability Promote left ventricular function	Facilitate eNOS-mediated Nrf2/Keap1 pathway to upregulate expression of HO-1 and GPX	Inhibitor	STZ-treated mice	Xiao et al., 2019
	GYY4137/hydrogen sulfide	Prevent MIRI Maintain the cardioprotection of SPC Relieve oxidative stress Reduce apoptosis Increase cardiac tissue viability	Decrease the level of PHLPP-1, inducing expression of AKT and Nrf2 Promote SIRT1/Nrf2 pathway	Inhibitor	STZ-treated mice	Qiu et al., 2018; Zhang J. et al., 2020
	Butin	Ameliorate MIRI Relieve oxidative stress Reduce apoptosis Lessen the infarct size	Facilitate AMPK/GSK-3β/Nrf2 signaling pathway	Inhibitor	STZ-treated mice	Duan et al., 2017

*CaSR, calcium-sensitive receptor; MuRF1, muscle-specific ring finger protein 1; GSK-3β, glycogen synthase kinase-3β; AKT, protein kinase B(PKB); SIRT1, silent information regulator 1; ARE, antioxidant response element; TGF-β, transforming growth factor-β; P62, a downstream Nrf2-driven gene; SIRT1KO mice, cardiac-specific SIRT1 knockout mice; PGC-1α, peroxisome proliferator-activated receptor gamma coactivator 1-alpha; eNOS, endothelial nitric oxide synthase; STZ, streptozocin; NF-κB, nuclear factor kappa B; MAPKs, mitogen-activated protein kinases; PHLPP-1, PH domain leucine-rich repeat protein phosphatase-1; FXR, farnesoid X receptor; NQO-1,NAD(P)H, quinone oxidoreductase 1; Prdx1, peroxide enzyme 1; iNOS, inducible nitric oxide synthase; EPO, erythropoietin; VEGF, vascular endothelial growth factor; IκBα, inhibitor of NF-κB α; ERα, estrogen receptor α; SPC, sevoflurane postconditioning; HMGCS2, 3-hydroxy-3-methylglutaryl-coenzyme A synthase 2; LKB1, liver kinase B1;Brg1, Brahma-related gene.*

Myocardial ischemia/reperfusion injury (MIRI) was more likely occur in diabetics, in addition to a worse prognosis, leading to myocardial vulnerability (Palomer et al., 2013). Under diabetic conditions, oxidative stress and programmed cell death were observed and supposed to turn down AMPK expression, conducing to a higher level of NADPH oxidase (Nox), whose main function is to supply ROS (Wang et al., 2020b). Also demonstrated is that suppressing ferroptosis may alleviate endoplasmic reticulum stress (ERS), which was triggered by ATF4-C/EBP homologous protein (CHOP) pathway and played an critical role in rat myocardial I/RI (Li W. et al., 2020). Some compounds have been approved to palliate MIRI; for example, honokiol (Zhang Y. et al., 2018), resveratrol (Xu G. et al., 2019), and luteolin (Xiao et al., 2019; Table 1), offering strong evidence of ferroptosis-dependent MIRI, which undoubtedly deserve further studies.

#### Vascular Injury

Current studies have shown that hyperglycemia-induced oxidative stress and incremental generation of ROS, two typical ferroptotic hallmarks, play capital roles in the development of endothelial dysfunction (Brandes and Kreuzer, 2005), which is a key contributor to diabetic vascular complications. The complications can be impressed by depressed production of NO, along with a growing level of inflammatory factors, endothelial repair dysfunction, and can contribute to the pathogenesis of thrombosis and AS (Dhananjayan et al., 2016; Luo et al., 2021). Past research identified particulate matter 2.5 (PM2.5) as a potential initiator of ferroptosis in endothelial cells due to its function of inducing ROS production and iron overload (Wang and Tang, 2019). New evidence has suggested that high glucose (HG) and interleukin-1β (IL-1β) treated with human umbilical vein endothelial cells (HUVECs) have ferroptosis-related characteristics, which were induced by augmenting the p53-system Xc-GSH pathway, and the same manifestation was aroused in the aorta of db/db mice as well (Luo et al., 2021). This evidence corroborates that ferroptosis is involved in endothelial dysfunction, though more experiments should be performed *in vivo*. Moreover, lipid oxidation is also linked to vascular smooth muscle cell (VSMC) injury, leading to vascular calcification. Studies have certified that increased FFAs can induce endothelial dysfunction and insulin resistance, prompting an intimate relationship with ferroptosis and VSMC calcification in patients with diabetes. It has been enunciated that metformin (Met) exerts a protective effect on VSMC calcification via its anti-ferroptotic role by boosting Nrf2 expression, and intriguingly, periostin (POSTN), known as an upregulated protein in AS, downregulates p53 to sensitize VSMCs to ferroptosis in this process (Ma et al., 2021). As previously discussed, anti-ferroptosis has a potential role against vascular calcification; meanwhile, both POSTN and p53 may become novel targets, revealing the secrets of prevention and treatment of vascular injury. Additionally, cigarette smoke extract (CSE) has the ability to trigger ferroptosis in VSMCs by consuming GSH, which cannot be reversed via increased GPX4; resulting in the fact alarming people that smoking is a high-risk factor of vascular injury (Sampilvanjil et al., 2020).

The accumulation of ROS, disturbance in lipid and glucose metabolism, endothelial dysfunction and vascular calcification are all-important pathogenic factors involved in AS (Gimbrone and Garcia-Cardena, 2016; Poznyak et al., 2020), which is characterized by lipid overload and the establishment of atherosclerotic plaques in the arterial wall (Falk, 2006). In addition, an enhancive level of iron has been examined in AS (Xu, 2019), and in a situation of iron overload, oxidative stress and endothelial dysfunction may aggravate the state of AS in apolipoprotein E knockout mice (Marques et al., 2019). This information motivated a research group to evaluate the inhibition of ferroptosis in AS with ferrostatin-1, in which they observed a positive therapeutic effect in both high-fat diet-fed mice and ox-LDL-treated mouse aortic endothelial cells (MAECs) (Bai et al., 2020a), thus unveiling the mysterious character of ferroptosis in AS. Generally speaking, prospective therapy for treating AS might be more attentive for the repression of ferroptosis and more comprehensive exploration of ferroptosis-related vascular injury is required.

### Ferroptosis and Metabolic Dysfunction-Associated Fatty Liver Disease (MAFLD)

To meet the demand of drug development and accurately reflect its mechanism, experts reached consensus to use the new terminology, metabolic dysfunction-associated fatty liver disease (MAFLD), to replace non-alcoholic fatty liver disease (NAFLD), which clearly put finger on the close link between this kind of chronic liver disease and metabolic disorder, such as diabetes, obesity and the metabolic syndrome (Eslam et al., 2020). It has been pointed out that free fatty acid accumulation, oxidative stress and inflammatory responses are all associated with MAFLD progression, however, the detailed mechanism propelling the simple steatosis in the directions to non-alcoholic steatohepatitis (NASH) still need to be enucleated (Mao et al., 2020). Of note, 4-hydroxy-2,3-non-anal (4-HNE), produced by lipid peroxidation reactions, may cause a damage to liver cells via Fenton reaction (Zhao et al., 2021) and it can be regarded as oxidative stress markers of NASH, so as the malondialdehyde (MDA) (Loguercio et al., 2001). Moreover, among all kinds of necrotic cell death, ferroptosis was verified to be the primary reason for the initiation of inflammation in NASH (Tsurusaki et al., 2019). Experiments have been carried out that interfering with FeCl3, specific responses of distinct liver-derived cells (rat primary hepatocytes (RPH), mouse primary hepatocytes (MPH), HepG2 human hepatoma cells and Hepa1-6 mouse hepatoma cells) were observed as a consequence of iron overload (Chen H. J. et al., 2020). Subsequently, Zhao et al. (2021) have described at length that GPX4 plays a key role in the protection of liver cells, indicating that targeting ferroptosis may be evolved into a new therapeutic options for MAFLD. Exerting a positive effect of preventing lipotoxicity, poly-(rC)-binding protein 1 (PCBP1), as a cytosolic iron chaperonin, delay the progression of ferroprosis in mouse liver (Protchenko et al., 2020). Sestrin2 (Park et al., 2019), Ginkgolide B (GB) (Yang et al., 2020) and dehydroabietic acid (DA) (Gao et al., 2021) became operative in relieving MAFLD mainly through activating Nrf2 pathway to obstruct ferroptosis while enoyl coenzyme A hydratase 1 (ECH1) (considered as an ingredient in mitochondrial fatty acid β-oxidation) seemingly play a role of mediating the Erk expression (Liu B. et al., 2021). Conducted in clinical trials, several antioxidants, such as Vitamin E and pioglitazone, are also proved to ameliorate oxidation levels resulting in the improvement of steatosis, inflammation, ballooning and fibrosis in NASH patients (Sanyal et al., 2010; Bril et al., 2019).

Remarkably, iron metabolism appears to have an intimate relationship with the disease development. Due to the metabolic disturbance, NASH is exacerbated as a result of hepatic iron deposition (Nelson et al., 2011) and can be improved by using deferoxamine mesylate salt (an iron chelator) and liproxstatin-1 (a ferroptosis inhibitor) in the methionine/choline-deficient diet (MCD) fed mice (Qi et al., 2020). In addition, the divalent metal transporter 1 (DMT-1) protein located in the small intestine is in charge of absorbing Fe^2+^ which is stored in cells subsequently and then exported by FPN1. Based on the level of iron and the saturation of FPN, hepatocytes take participated in modulating iron homeostasis through secreting hepcidin which can attenuate the production of DMT-1 in order to regulate the absorption of Fe^2+^. A research showing that serum hepcidin level and expression of DMT1 messenger RNA were upregulated depending on the enhancive activity of iron regulatory protein (IRP) in the NASH patients during an oral iron absorption test (OIAT) (Hoki et al., 2015), which was consistent with the observations of previous studies (Senates et al., 2011). Most recently, Yu et al. (2020) have clarified that ferroptosis-induced liver fibrosis may easily develop in hepatocyte-specific Trf (encoding transferrin) knockout mice (Trf-LKO) with a high-iron diet, which can be reversed with the treatment of reducing Slc39a14 expression in liver or applying ferrostatin-1.

### Ferroptosis and Osteoporosis

Accumulating researches have demonstrated that inordinate iron metabolism can destroy bone homeostasis inducing osteoporosis, which is a systemic metabolic disease characterized by reduced bone mass, augmented bone fragility and increased risk of fracture (Che et al., 2020). Conducted within certain iron overload models, the phenotype of diminished bone density and trabecular thickness was observed due to heightened osteoclast differentiation and osteoblast apoptosis, leading to intense bone dissolution and abating bone formation due to decreased osteoblast specific genes, i.e., alkaline phosphatase (ALP), runt-related transcription factor 2 (Runx2) and type I collagen (Tsay et al., 2010; Li et al., 2012; Chen et al., 2014; Cheng et al., 2017). Redundant iron stimulates osteoclasts and osteoblasts to yield a generous amount of ROS, engendering maladjustment of the intracellular antioxidant/peroxidation balance system by activating the MAPKs and NF-κB pathways, resulting in osteoblast death (Ray et al., 2012; Che et al., 2020). All of the above discoveries foreshadow that ferroptosis may participate in the development of osteoporosis, especially the devitalization of osteoblasts. Recently, a study indicated that extracellular vesicles derived from endothelial progenitor cells (EPC-EVs) satisfied the need of retarding the progress of steroid-induced osteoporosis (SIOP) by inhibiting ferroptosis, specifically by reversing the deactivation of GPX4, system Xc– and decreased cysteine levels, which is initiated by dexamethasone (Lu et al., 2019). Since these speculations are based on bioinformatics evidence, the detailed signal pathways and *in vitro* and *in vivo* experiments warrant further investigation. Another study sustained these results. Due to the ferroptosis-induced role played by glucocorticoids in osteoblasts (Lu et al., 2019), Yang et al. (2021) originally found that exosomes derived from vascular endothelial cells (EC-Exos) counteract this process both *in vitro* and *in vivo* by decreasing NCOA4 expression to suppress ferritinophagy and simultaneously targeting the Keap1-Nrf2-HO-1/NQO-1 pathway to inhibit lipid oxidation, though the specific signal has not yet been elucidated.

Some drugs that target osteoporosis may actualize a ferroptotic role during the therapeutic mechanism, which, in our perspective, is reasonable to surmise, but not yet visible. Artemisinin (ARS) and its related compounds, which are already known as inhibitors of osteoporosis due to their suppression of osteoclast differentiation by blocking the receptor activator of nuclear factor kappa-B ligand (RANKL) pathway, may induce ferroptosis in osteoclasts due to the high levels of ferritin and LIP in osteoclasts (Zhang, 2020). Furthermore, andrographolide (AP), an herbal medicine, was found to have the ability to regulate the osteoprotegerin (OPG)/RANKL signal (Tantikanlayaporn et al., 2020) and stifle the NF-κB pathway activated by TNFα (Yongyun et al., 2019) so as to hasten osteoblast differentiation. It may also weaken the extracellular signal-regulated kinase (ERK)/MAPK and NF-κB signals in order to block RANKL-induced osteoclast differentiation (Yongyun et al., 2019). Engagingly, those signal ways are also targeted in the protection of diabetic myocardial dysfunction (Table 1), which naturally evokes reflections about whether a drug can work on the Nrf2/ARE pathway as well exert an antioxidant effect to inhibit ferroptosis in osteoporosis. Compounds such as luteolin (Jing et al., 2019), isoliquiritigenin (Jeong et al., 2020), and myricitrin (Huang et al., 2014) may play a corresponding role as andrographolide, which may promote or inhibit ferroptosis in osteoclasts and osteoblasts alike. Remarkably, Ma H. et al. (2020) first reported that melatonin is capable of restraining ferroptosis in T2DM-induced osteoporosis, mainly by accelerating the Nrf2/HO-1 pathway, and provided convincing evidence to motivate extensive work to be done in the future.

In conclusion, the stimulation of ferroptosis in osteoclasts and its converse repression in osteoblasts in the prevention and treatment of osteoporosis may be a potential field of exploration. Likewise, some drugs affecting osteoporosis appear to have the latent target to Nrf2 or to the other key mediators of ferroptosis, which have maintained undetected.

### Ferroptosis and Other Metabolic and Endocrine Disorders

There also exist some researches of the role of ferroptosis in endocrine gland disorders. Some studies, which examined the relationship between adrenal gland and ferroptosis, suggested that a adrenocortical cell-producing steroid presents a higher sensitivity to ferroptosis, compared to the non-steroidogenic adrenal medulla, when the GPX4 is suppressed, mainly because of its elevated expression of GPX4, ACSL4, adrenic and arachidonic acid, which are key regulators of ferroptosis (Weigand et al., 2020). Adrenocortical carcinomas (ACCs) are also sensitive to the induction of ferroptosis, and based on this result, a new therapeutic target to ACCs may present beyond the mitotane who cannot energize the ferroptosis pathway (Belavgeni et al., 2019). Additionally, due to prostate cancer cells’ high sensitivity to iron toxicity, activating ferroptosis appears to be a new strategy for treating advanced prostate cancer, with an option to include second-generation anti-androgens (Bordini et al., 2020; Ghoochani et al., 2021). As an FDA-approved anthelmintic, flubendazole was found to target P53 to induce ferroptosis in an effort to delay the development of prostate cancer (Zhou et al., 2021). At present, ferroptosis-related work in endocrine gland disorders mostly focus on cancer cells; the link between other types of diseases and ferroptosis is still unknown.

Interestingly, level of hormone is closely correlated to regulations of cell growth. A recent study suggested that hyperandrogenism and insulin resistance may trigger the gravid uterine and placental ferroptosis in rats with polycystic ovary syndrome (PCOS), leading to fetal loss (Zhang Y. et al., 2020). Actually, it is acknowledged that a variety of hormones, such as melatonin (Dehdashtian et al., 2018), thyroid hormone (Liu et al., 2019), glucocorticoid, estrogens and androgens (Billig et al., 1996), among others, could bring about an effect to control cell death, causing autophagy, inflammation, oxidative stress and cancer progression. Questions remain; for instance: does ferroptosis have a share in this process?; will a curative effect result if using inhibitors against ferroptosis in cells and tissues undergoing hormone hypersecretion? All of these issues need to be researched further. Ferroptosis was also supposed to act on some specific inherited metabolic diseases such as lysosomal storage diseases (LSD), which was reviewed integrally by Pierzynowska et al. (2021) from a perspective of mechanism in autophagy-dependent ferroptosis. Moreover, with the discovery of transferrin receptor 1 downregulating in satellite cells of old mice, researchers caught sight of the relationship of ferroptosis and age related impairment of skeletal muscle regeneration gradually (Ding et al., 2021). Overall, to date, many illuminating reviews on ferroptosis from different perspectives have emerged, providing a much wider view for researchers to its mechanism and treatment for the related diseases (Jiang et al., 2021). Despite some of the way ahead still shrouding in mist, the research into ferroptosis is in full swing and a rapid progress has been made as more and more metabolic diseases having been found to possess ferroptotic-feature (Conrad et al., 2021).

## Conclusion and Outlooks

In this review, we summarized three essential conditions and specific mechanisms of ferroptosis development, including three major regulated pathways that repaired lipid oxidation disturb, which may cause ROS accumulation, oxidation of PUFAs and excessive iron. We concluded the emerging regulators targeted the key mediators of ferroptosis systematically and from all sides, assisting readers to comprehend the latest investigations in the field of ferroptosis. Moreover, we placed emphasis on the ferroptotic role in some metabolic disorders, offering an intimate portrait of diabetes and its complications and osteoporosis.

In recent years, there has been a heated discussion regarding ferroptosis in the academic world, since it is a novel type of RCD involved in many diseases. Seldom could anyone deny the importance of ferroptosis in cell growth and its role in the prevention and treatment of diverse disorders. However, from our perspective, there are still some problems pressed for solutions. Firstly, is there a synergetic or antagonistic effect between ferroptosis and the other forms of RCD as they share some critical regulators in the pathway (such as P53)? Secondly, the detailed molecular mechanisms that triggered ferroptosis still present with some void. For instance, the downstream effectors of lipid oxidation have not yet been identified and the level of iron and ROS that should be achieved to initiate ferroptosis remain unknown. Besides, the study about the GCH1-BH4-phospholipid axis that was recently published is still in its infancy. The other mediators targeted by this pathway should be investigated. Some of the existing studies results are ambivalent; for example, the precise role of Nrf2 in the induction of ferroptosis; specifically, whether activating or blocking Nrf2 appears to have a dual effect on diabetes and its complication, which are conducted with same method in different laboratories. In addition, does ferroptosis definitely occur in cells possessing the three necessary conditions? What is the alternative requirement of cells to improve or reduce the sensitivity to ferroptosis? Thirdly, as previously mentioned, exosomes may participate in the regulation of ferroptosis, not only exporting iron out of cells to prevent ferroptosis, but also reversing peroxidation through the delivered effect. This effect is performed by the exosomes derived from some certain types of cells, such as endothelial progenitor cells. Therefore, in the long run, the crosstalk between exosomes and ferroptosis may be a future hot topic. Last but not least, with the shortage of human evidence, there is a long way to go in terms of applying basic research results of ferroptosis to clinical applications. This brings into question if making use of ferroptotic inhibitors will do harm to other cells and tissues that rely on the metabolism of iron and ROS, or from a different angle, if anti-ferroptotic compounds present as the antioxidant agents to be applied to some metabolic diseases caused by oxidative stress.

In summary, ferroptosis is undoubtedly regarded as a promising target to treat metabolic diseases, however, the complete molecular mechanism and its underlying role in metabolic diseases still warrant further examination.

## Author Contributions

L-QY wrote the manuscript and approved the final version of the manuscript. J-YD contributed to study conduct, data analysis, and manuscript writing. XL, FX, S-KS, BG, F-X-ZL, M-HZ, YW, Q-SX, L-ML, W-LO-Y, Y-YW, and K-XT contributed to data analysis. All authors reviewed the manuscript.

## Conflict of Interest

The authors declare that the research was conducted in the absence of any commercial or financial relationships that could be construed as a potential conflict of interest.

## Abbreviations

RCD: regulated cell death
AS: atherosclerosis
ACD: accidental cell death
ROS: reactive oxygen species
MAPKs: mitogen-activated protein kinases
GPX4: glutathione-glutathione peroxidase 4
GSH: glutathione
PUFAs: polyunsaturated fatty acids
TF: transferrin
TFR1: transferrin receptor 1
LIP: labile iron pool
FPN1: ferroportin 1
LCN2: lipocalin 2
NCOA4: nuclear receptor coactivator 4
NUPR1: transcriptional regulator
PCBP1: poly-(rC)-binding protein 1
HO-1: heme oxygenase-1
PE: phosphatidylethanolamine
AA: arachidonic acid
ACSL4: Acyl-CoA synthetase long-chain family member 4
LPCAT3: lysophosphatidylcholine acyltransferase 3
LOX: lipoxygenase
POR: cytochrome P450 oxidoreductase
CYB5R1: cytochrome b5 reductase
PLOOH: phospholipid hydroperoxide
AGPS: alkylglycerone phosphate synthase
FAR1: fatty acyl-CoA reductase 1
GNPAT: glyceronephosphate O-acyltransferase
AGP: 1-O-alkyl-glycerol-3-phosphate
AGPAT3: 1-acylglycerol-3-phosphate O-acyltransferase 3
PEDS1: plasmanylethanolamine desaturase 1
L-OOH: lipid hydroperoxides
L-OH: lipid alcohols
Nrf2: (Erythoid-derived)-like 2
Keap1: Kelch-like ECH-associated protein 1
T1DM: Type 1 diabetes
T2DM: Type 2 diabetes
ARF: CDKN2A locus
ATF3: transcription factor 3
BAP1: BRCA1-associated protein 1
PDK4: pyruvate dehydrogenase kinase 4
CD44v: CD44 variant
LAT1: L-type amino acid transporter 1
Sec: selenocysteine
IPP: isopentenyl pyrophosphate
HSPA5: heat shock protein family 5
Se: selenium
H2O2: hydrogen peroxide
FSP1: ferroptosis suppressor protein 1
AIFM2: apoptosis-inducing factor mitochondrial 2
NADPH: nicotinamide adenine dinucleotide phosphate
CoQ10: ubiquinone
MDM2: murine double minute 2
PPAR α: peroxisome proliferator-activated receptor α
MEG3: maternally-expressed gene 3
ATF4: transcription factor 4
BAT: brown adipose tissue
FFA: free fatty acids
ALA: alpha lipoic acid
DRG: dorsal root ganglion
DN: diabetic neuropathy
UCP2: uncouplingprotein 2
GCH1: GTP cyclohydrolase-1
BH4/BH2: tetrahydrobiopterin/dihydrobiopterin
RTAs: radical-trapping antioxidants
DHFR: dihydrofolate reductase
NOS: nitric oxide synthase
Ox-LDLs: oxidized low-density lipoproteins
AMPK: AMP-activated protein kinase
RNS: reactive oxygen and nitrogen species
FIA: ferroptosis-inducing agents
CCA: cryptochlorogenic acid
Fer-1: ferrostatin-1
HIF: hypoxia-inducible factor
HMGB1: high-mobility group box-1
DCM: diabetic cardiomyopathy
HSF1: heat shock factor 1
CaSR: calcium-sensitive receptor
MuRF1: muscle-specific ring finger protein 1
SFN: sulforaphane
MT: metallothionein
MIRI: myocardial ischemia/reperfusion injury
Nox: NADPH oxidase
ERS: endoplasmic reticulum stress
CHOP: ATF4-C/EBP homologous protein
PM2.5: particulate matter 2.5
IL-1 β: interleukin-1 β
HG: high glucose
HUVECs: human umbilical vein endothelial cells
VSMC: vascular smooth muscle cell
Met: metformin
POSTN: periostin
CSE: cigarette smoke extract
MAECs: mouse aortic endothelial cells
ALP: alkaline phosphatase
Runx2: runt-related transcription factor 2
SIOP: steroid-induced osteoporosis
EPC-EVs: endothelial progenitor cells derived extracellular vesicles
EC-Exos: endothelial cells derived exosomes
ARS: artemisinin
RANKL: nuclear factor kappa- B ligand
AP: andrographolide
OPG: osteoprotegerin
ERK: extracellular signal-regulated kinase
ACCs: adrenocortical carcinomas
PCOS: polycystic ovary syndrome
ATM: Ataxia-Telangiectasia
MTF1: metal-regulatory transcription factor 1
MAFLD: metabolic dysfunction-associated fatty liver disease
NAFLD: non-alcoholic fatty liver disease
NASH: non-alcoholic steatohepatitis
4-HNE: 4-hydroxy-2,3-non-anal
MDA: malondialdehyde
RPH: rat primary hepatocytes
MPH: mouse primary hepatocytes
GB: Ginkgolide B
ECH1: enoyl coenzyme A hydratase 1
MCD: methionine/choline-deficient diet
DMT-1: divalent metal transporter 1
IRP: iron regulatory protein
OIAT: oral iron absorption test
LSD: lysosomal storage diseases.
